# It Is Our Turn to Get Cannabis High: Put Cannabinoids in Food and Health Baskets

**DOI:** 10.3390/molecules25184036

**Published:** 2020-09-04

**Authors:** Seyed Alireza Salami, Federico Martinelli, Antonio Giovino, Ava Bachari, Neda Arad, Nitin Mantri

**Affiliations:** 1 Faculty of Agricultural Science and Engineering, University of Tehran, Karaj 31587, Iran; 2 Department of Biology, University of Florence, Via Madonna del Piano, 6, Sesto Fiorentino, 50019 Firenze, Italy; federico.martinelli@unifi.it; 3 Council for Agricultural Research and Economics (CREA), Research Centre for Plant Protection and Certification (CREA-DC), 90011 Bagheria (PA), Italy; antonio.giovino@crea.gov.it; 4 School of Science, RMIT University, Melbourne, Bundoora, VIC 3083, Australia; ava.bachari@student.rmit.edu.au (A.B.); nitin.mantri@rmit.edu.au (N.M.); 5 School of Plant Sciences, The University of Arizona, Tucson, AZ 85721, USA; nedaarad@email.arizona.edu

**Keywords:** hemp, phytocannabinoids, receptors, CNS, PNS, gene networks, CBD oil, medcannabase

## Abstract

Cannabis is an annual plant with a long history of use as food, feed, fiber, oil, medicine, and narcotics. Despite realizing its true value, it has not yet found its true place. Cannabis has had a long history with many ups and downs, and now it is our turn to promote it. Cannabis contains approximately 600 identified and many yet unidentified potentially useful compounds. Cannabinoids, phenolic compounds, terpenoids, and alkaloids are some of the secondary metabolites present in cannabis. However, among a plethora of unique chemical compounds found in this plant, the most important ones are phytocannabinoids (PCs). Over hundreds of 21-22-carbon compounds exclusively produce in cannabis glandular hairs through either polyketide and or deoxyxylulose phosphate/methylerythritol phosphate (DOXP/MEP) pathways. Trans-Δ9-tetrahydrocannabinol (Δ9-THC) and cannabidiol (CBD) are those that first come to mind while talking about cannabis. Nevertheless, despite the low concentration, cannabinol (CBN), cannabigerol (CBG), cannabichromene (CBC), tetrahydrocannabivarin (THCV), cannabidivarin (CBDV), cannabinodiol (CBND), and cannabinidiol (CBDL) may have potentially some medical effects. PCs and endocannabinoids (ECs) mediate their effects mainly through CB1 and CB2 receptors. Despite all concerns regarding cannabis, nobody can ignore the use of cannabinoids as promising tonic, analgesic, antipyretic, antiemetic, anti-inflammatory, anti-epileptic, anticancer agents, which are effective for pain relief, depression, anxiety, sleep disorders, nausea and vomiting, multiple sclerosis, cardiovascular disorders, and appetite stimulation. The scientific community and public society have now increasingly accepted cannabis specifically hemp as much more than a recreational drug. There are growing demands for cannabinoids, mainly CBD, with many diverse therapeutic and nutritional properties in veterinary or human medicine. The main objective of this review article is to historically summarize findings concerning cannabinoids, mainly THC and CBD, towards putting these valuable compounds into food, feed and health baskets and current and future trends in the consumption of products derived from cannabis.

## 1. Introduction

Over 350,000 plant species have been identified worldwide among which about 10–20% (35,000 to 70,000 plant species) are used for medicinal purposes. Apart from their medicinal use, plants are also important for nutrition (human and animals), can be used as building materials, and are important sources of fiber, dyes, and spices [1]. Herbal medicine has been associated with human life since the beginning. There are number of ailments for which there is currently no cure. The use of medicinal plant extracts either alone or in combination with allopathic drugs is currently used to treat a number of diseases and disorders [2]. Indeed, the World Health Organization (WHO) estimated that more than 80% of the world’s population in developing countries depend on herbal medicine to meet their basic health needs [3]. Therefore, the use of traditional medicine is critical to combat various diseases and disorders.

Among the medicinal herbs known, some, such as poppy and cannabis, have a long history of use. Cannabis is both a medicinal and industrial plant native to Central Asia. It has been globally used for thousands of years as a source of food, feed, fuel, fiber, medicine, and narcotics [4,5,6].

There is a growing demand for compounds called “cannabinoids” with many diverse magic therapeutic properties. Pharmaceutical science has discovered an ancient herb with promising effects which may save our planet in the third millennium.

During more than 6000 years of cannabis emergence, over 25,000 different products have been derived and used for various purposes from this plant. Long after using fiber and seeds, cannabis was used as a medicine from the time of Emperor Shen-Nung (2.700 B.C.). In ancient medicine, cannabis was used to treat rheumatic pain, intestinal constipation, female reproductive disorders, and malaria [7,8]. Drug-type cannabis is generally called marijuana, while industrial cultivars are called hemp. Presently, there is an intense scientific competition between the world’s largest research centers in the quest for finding cannabis plants with unique specific genotypes/chemotypes and hemp varieties with low THC (<0.2–0.3%) that can be industrially used or cultivated. Cannabis can be fibrous in nature and can be easily cultivated on a large scale and used as an industrial material. Accordingly, the figures indicate an annual trade of over USD 100 million for these chemotypes [9,10]. According to the United Nation Office on Drugs and Crime, cannabis is being the most widely used illegal drug of the 21st century [11]. Given the nutritional and medicinal value of cannabis and the urgent need to provide appropriate medicines for the treatment of major diseases worldwide, such as multiple sclerosis (MS), cancer, and AIDS, the natural medicinal potential of using this plant for disease treatment requires further exploration. In addition, as a result of the well-recognized negative effects of some synthetic drugs and the tendency of physicians and patients to prescribe and use safe and effective drugs, there is an increasing need for the use of safe herbal resources with little or no side effects. Consequently, the demand for research on the discovery of active ingredients of medicinal plants such as cannabis for medical applications has been increasing too. As a result, its genome and transcriptome have now been sequenced [12]. Some of the most effective drugs for the prevention or treatment of major diseases such as malaria, MS, etc. in humans have been derived from medicinal plants. This has also stimulated the interest of researchers with respect to medicinal plants especially on the therapeutic effects of the resulting compounds extracted from these plants.

The compounds obtained from cannabis can be used to treat pain and alleviate the side effects of some medical treatments. For example, cannabidiol, one of the most important cannabis compounds, relieves all kinds of chronic and nonchronic pain and can be used to treat febrile convulsion, inflammation, anxiety, and nausea [13]. Recent studies have shown that these compounds are effective in treating breast and other forms of cancers and in modulating or relieving the symptoms of schizophrenia, multiple sclerosis (MS), migraine, and rheumatism [14]. Marinol^®^ is another important cannabis-derived drug which is prescribed as an appetite suppressant and an antinausea medication for people undergoing chemotherapy or suffering from radiation-induced nausea or neurodegeneration due to AIDS. The positive effects of cannabis and its cannabinoids have been shown to be effective in partially alleviating the symptoms of AIDS [15]. Over the past decade, there has been a concerted effort to reduce deforestation and promote the need and use of cannabis and hemp in different industries. This has become more important given the rediscovery of the magical properties of plants such as cannabis in treating or alleviating the symptoms of many different diseases including cancer and MS.

Cannabis is not popular just for its narcotic and therapeutic properties but also for many other positive effects on human life such as fiber-based products, textiles, nutritional supplements and seed oil, food and feed, and cosmetics. Cannabis (hemp) has been known for many centuries as an excellent source of fiber because of the cheap processing costs and the high quality of cannabis fibers. The strongest and longest natural fibers are obtained from cannabis with fiber extraction being one of the most important uses of hemp. Fiber is used as a raw material in the manufacture of ropes, sails, and fabric [16]. It is also stronger and softer than cotton and other similar textiles that are typically used in today’s manufacturing processes. Cannabis fiber has been used for many years as a safe, inexpensive, environmentally friendly, and recyclable raw material for the production of paper, paper towels, clothing and hats, and even shoes in many countries in Europe, especially in the Netherlands. Since the hemp is very rich in cellulose, it is now increasingly being used in the production of degradable plastic products. Hemp fibers are also now used as a component of fiberglass production in many industries. Given the pressing need to reduce deforestation and the need for the use of renewable fiber in the wood and paper industries, hemp is a good candidate for promoting the efficient use of natural resources from forests. Despite the decline in cannabis cultivation due to existing legal constraints, some EU policies have been developed to encourage the cultivation of hemp as a natural fiber producing plant species [17].

Hempseeds have also been used by humans for a long time as a rich source of protein and oil. Hempseeds are one of the most nutritious seeds in the world containing 20–25% protein, 20–30% carbohydrate, 25–35% oil, and 10–15% insoluble fiber. These seeds also contain optimum amounts of phosphorus, potassium, magnesium, sulfur, calcium, iron, zinc, and a variety of vitamins A, C, and E as well as minerals, beta-carotene, and antioxidants [18]. The hempseed oil contains balanced ratios of optimum amounts of unsaturated chain fatty acids, including linoleic acid and linolenic acid, which are beneficial to human nutrition and health and reduce cholesterol levels and hypertension [19]. Additionally, due to the high amounts of PUFA and the presence of gamma-linoleic acid, the hempseed oil is used in the cream and lacquer manufacturing industries and in cosmetics such as moisturizers and shampoos, massages oils, conditioners, and lotions. Cannabis health products are not only allergenic, but they also have skin repair properties. At the same time, these products are free of chemicals and toxins that are sometimes used in most synthetic cosmetics. Besides antibiotic properties, CBD and THC have painkiller and anti-inflammatory components, respectively. Therefore, a mixture of cannabinoids may produce a tattoo ink with a low risk of infection and inflammation [20].

Cannabis is also used for the preparation of two important biofuels—biodiesel and bioethanol. Many green or environment-friendly vehicles nowadays work with a variety of biofuels such as corn oil and bioethanol derived from plants. The limited fossil fuels reserves, their comparatively higher cost of extraction, and the disadvantages of using them (pollution of the environment) have led people to focus on the use of renewable energies. Hemp fuel can be a good alternative to gasoline. The hydrocarbons in this plant can be burnt as a renewable and nonpolluting source of the atmosphere and used in the production of alternative fuels such as ethanol and biodiesel.

Cannabis has had a long history with many ups and downs. Now, it is our turn to get the cannabis high. The main objective of this review article is to historically summarize findings concerning cannabinoids, mainly THC and CBD, towards putting these valuable medical compounds into food and health baskets and current and future trends in the consumption of products derived from cannabis/hemp. The cannabinoid biosynthesis pathway is discussed.

## 2. Cannabis Botany and Its Constituents

*Cannabis sativa* L. is an herbaceous annual plant belonging to the family Cannabacea [21], and the plant varieties have a chromosomal system that has been designated as X and Y (chromosomes) [22]. The genus *Cannabis* has four species namely, *C. sativa*, *C. indica*, *C. ruderalis*, and *C. afghanica* [23]. The male and female flowers of this bipedal plant are on separate bases with male flowers generally being formed earlier than female flowers [21]. Male flowers are formed at the end of the stem, complex in nature, and pendulous on the base outside the leaves congress. Each of these flowers also has five flags inside the flower cover. The female flowers generally look the same as the male flowers, except that they resemble leaf-like brackets without a distinct demigod. The fruit of this plant is dark brown and has an albumin-free seed. Cannabis plant contains different types of phytochemicals with a diverse range of effects [24,25,26]. Some of these compounds belong to primary metabolites while others are secondary metabolites and at the center of attention of the food, feed, and health industries.

## 3. Cannabis Secondary Metabolites

Cannabis plants contain approximately 600 identified and many yet unidentified potentially useful compounds [24,25]. The main cannabis secondary metabolites include cannabinoids, phenolic compounds such as flavonoids, stilbenoids, lignamides, and phenolic amides, terpenoids, alkaloids, noncannabinoid phenols, as well as other common compounds [25,27]. However, the most important secondary metabolites from cannabis are the cannabinoids. This group of compounds is found exclusively in cannabis and has been the most thoroughly characterized chemical substances in this plant. Production of such compounds depends on cultivar genetic background, plant developmental stage and maturity, growth conditions, harvest time, storage and poststorage conditions [24,27,28,29] and also environmental stress [30], ecological interactions between cannabis and ecosystem factors [23], and elicitors [31].

Here, we present a summary and review of the characteristics of secondary metabolites of cannabis with a special focus on cannabinoids.

## 4. Phenolic Compounds: Flavonoids, Stilbenoids, Lignamides, and Phenolic Amides

Phenolic compounds consist of more than 10,000 diverge ranges of molecules such as phenolic acids, flavonoids, stilbenes, and lignans [25]. Flavonoids are one of the major ubiquitous compounds in plants including cannabis which regulate very important and crucial roles [32]. Over 20 types of flavonoids have been reported in cannabis [21,33,34]. These flavonoids have been isolated from leaves, flowers, and pollens and include the *O*-glycoside versions of the aglycones apigenin, luteolin, orientin, vitexin and isovitexin, kaempferol and quercetin, as well as cannflavin A and cannflavin B, which are methylated isoprenoid flavones that are unique to *Cannabis* [21,35]. The flavone and flavonol biosynthesis in cannabis were represented by Flores-Sanchez and Verpoorte, 2008. Phenylalanine and malonyl-CoA are two precursors which trigger the enzymatic synthesis cascade of main structures of flavones and flavonols in cannabis [21].

Stilbenoids are another group of phenolic compounds with significant roles in plant defense [36,37], growth inhibition [38] and insect repellent [39], and also exhibit medicinal properties such as antioxidant activity [40] antibacterial and antifungal activities [41,42]. Three new stilbenoids (HM1, HM2 and HM3) and 19 known stilbenoids (Dihydroresveratrol; canniprene; cannabistilbene I, IIa, and IIb; cannithrene; cannabispirone; cannabispirenone, etc.) were isolated from cannabis [43,44]. These stilbenoids have been found in stems [45], leaves [46], and resins [47].

Cannabis also produces other phenolic compounds called phenolic amides and lignamides include cannabisin and cannabisinlike compounds as well as grossamide [21,48].

## 5. Terpenoids

Terpenoids are another major group of plant metabolites seen in cannabis. Along with their role in photosynthesis, respiration, and plant defense, terpenoids are responsible for the flavor and aroma of different cannabis varieties and are considered as a major crucial factor of preference of the cannabis users. The therapeutic benefit of medicinal cannabis is that to produce a retinue effect, cannabinoids may function in synergy with terpenoids [49]. Over one hundred monoterpenes and sesquiterpenes have been identified in cannabis [50]. These flavonoids have been isolated from roots, leaves, female flowers, and trichomes and include limonene, safranal, geraniol, α-curcumene, α-selinene, and farnesol [28,51,52,53]. The terpenoids biosynthesis pathway is initiated from isopentenyl diphosphate (IPP) and its isomer dimethyl-allyl diphosphate (DMAPP) which is derived from the cytosolic mevalonate pathway (MVA) or plastidial deoxyxylulose phosphate/methyl-erythritol phosphate (DOXP/MEP) pathway and ended up with different types of C10, C15 and C30, C20 compounds [21].

## 6. Alkaloids

Since morphine discovery in poppy, ~20,000 alkaloids identified in different species have been used as pharmaceuticals, stimulants, narcotics, and poisons [54,55]. Alkaloids are another class of secondary metabolites that have been reported in cannabis [43]. These extraordinary organic secondary metabolites have a wide range of bioactivities serving as end products of metabolism or waste products, storage reservoirs of nitrogen, protective agents against predators and stresses, pyridine nucleotide cycle which supplies the cofactor NAD, and as the precursor of the membrane phospholipid phosphatidylcholine [56,57,58]. Different types of alkaloids such as choline, neurine, L-(+)-isoleucine-betaine and muscarine, hordenine and trig pyrrolydine, onelline, cannabisativine, and anhydrocannabisativine were isolated from roots, leaves, stems, pollen, seeds, and hairy roots [21,59,60,61,62]. The alkaloids family comprises a large number of basic nitrogenous compounds with a complex biosynthesis pathway which was previously well described [21,63].

## 7. Phytocannabinoids

Cannabis is known as a complex multipurpose plant with a long history of use as a medicine, drug, and source of fiber [12,21,25]. Among a plethora of unique chemical compounds found in cannabis, the most important secondary metabolites are cannabinoids which are the most studied compounds in this plant. There are a growing number of articles that explore the importance of compounds called “cannabinoids’’ exclusively found in *Cannabis* plants. Phytocannabinoids are 21-22-carbon compounds, classified into 10 structural types with over 100 varieties occurring in resin produced by glandular hairs of *Cannabis sativa* L. [21,25,50,64,65]. Among these cannabinoids, trans-Δ9-tetrahydrocannabinol (Δ9-THC) and cannabidiol (CBD) are the two which have been the subject of more than 90 percent of scientific investigations. Although THC is the first thing that comes to mind when talking about cannabis, there are also nonpsychoactive cannabinoids with several therapeutic properties such as CBD, which has recently become more important due to THC limitations and new discoveries about CBD medicinal properties in parallel. Indeed, the plant’s adverse psychotropic and addictive effects are attributed to tetrahydrocannabinol [21,50,66]. The use of cannabis has always been very controversial because it is classified as an illegal drug due to its psychoactive THC. Female flowers (marijuana) and leaves (grass) of drug-type cannabis are considered as a “soft” drug but not “Hash or Hashish” which contains a high concentration of THC with harmful effects [67]. Despite this fact, there has been an increased interest in recent years of the potential use of cannabis derivatives in medical applications [65]. In this regard, reviewing and rewriting the regulations by governments, introducing novel gene pools of dwarf and low THC/high CBD accessions, and the development of techniques to accurately characterize the chemical phenotypes (drug and nondrug cannabis plants) have made a significant contribution to the acceptance of this issue and the control of legal and illegal cannabis cultivation [6,68].

Five chemotypes were defined based on phytocannabinoids composition, quantities, and their ratios to discriminate different cannabis varieties for different applications [69,70,71,72,73]. Based on the THC content, cannabis chemotypes can be classified as drug type (marijuana, 1–20% THC), intermediate type (0.3–1.0% THC), and fiber type (hemp, <0.3% THC) [74,75]. Of course, this is only a widely accepted agreement to determine the restrictions of cultivation in different countries, however, based on our recent screening program, we found accessions in our gene bank that have unique fiber anatomy but contain >1% THC (CGRC, www.medcanabase.org). However, given that THC and CBD levels are more focused largely to determine whether it is a drug-type, fiber-type, or medical cannabis. According to this, a novel native cannabis gene pool was published recently at the Center for Genetic Resources of Cannabis (CGRC, www.medcanabase.org) with three principal chemotypes as follow: THC-chemotype I (high THC/CBD or low CBD/THC ratio, THC content over 0.3–20%, and CBD content lower than 0.5%); an intermediate-chemotype II (the concentration of both THC and CBD are in a ratio close to the unity mainly 0.5–2); CBD-chemotype III (high CBD/THC or low THC/CBD, has mainly CBD, an amount of THC lower than 0.3% to trace an undetectable level). Two other tentative additional chemotypes included chemotype IV which had CBG as the predominant cannabinoid, but also CBD was present, and chemotype V, which referred to a material with undetectable amounts of any cannabinoids, was qualitatively described by some authors. Medical varieties cover both THC and CBD chemotypes. The THC-chemotype, which may be termed marijuana, is defined as a psychotropic or medicinal type (based on THC level) and CBD-chemotype, which termed hemp as a “fiber”, “non-drug” type or “medical”.

**Figure 1:**
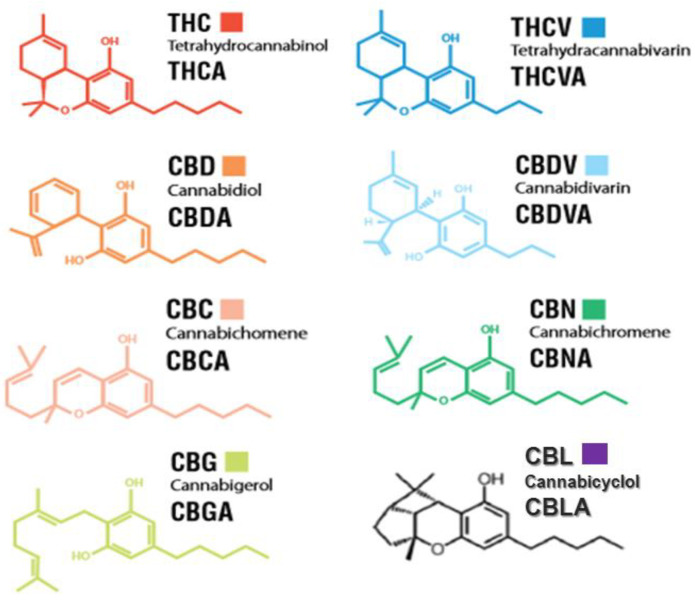
Natural important cannabinoids and their structure.

THC and CBD are at the center of others’ attention. It is a wonder why we have never heard too much of the other cannabinoids. Some of other well-known phytocannabinoids are shown in Figure 1, which may have possible medical effects including cannabinol (CBN), cannabigerol (CBG), cannabichromene (CBC), tetrahydrocannabivarin (THCV), cannabidivarin (CBDV), and cannabinodiol (CBND), cannabinidiol (CBDL), cannabielsoin (CBE), cannabitriol (CBT), cannabicyclol (CBL), and other minor compounds (Figure 1). Some of these cannabinoids such as THC, CBN, and CBDL are psychoactive, whereas CBD, CBC, and CBG are not [21,50,76,77,78].

Our knowledge of these compounds is less than that of our moon! Due to some potent sedative properties, cannabinol (CBN) which is found in both THC-rich cannabis and hemp is a little more known. CBN production is more and less due to an age-related degradation process which converts THC to CBN. CBN is also produced when THC is heated or exposed to oxygen [79].

Cannabigerol (CBG) is one of the most important cannabinoids you have just heard about as the precursor to both THC and CBD perhaps due to its low concentration and rare trace. Indeed, it devotes itself entirely to creating THC and CBD so that we can get high and/or enjoy good health and aspirations. However, CBG is promising in therapeutic applications, although most strains often contain less than 1% CBG. CBG is found rarely in low THC and CBD hemp cultivars but probably not in THC-rich cannabis and CBD-rich hemp cultivars.

Cannabichromene (CBC) is currently being researched for its medicinal benefits and has been found in tiny amounts in both THC-rich cannabis and CBD-rich hemp. CBC is also considered among the most promising high potential cannabinoids in recent medical research. CBC is the end-product of cannabidiol acid (CBDA) when it is broken down by heat or UV.

The importance and reputation of cannabidivarin (CBDV) is linked to its role in neurological disorders [80] and ongoing research on the antidiabetic effects of THCV [77,81]. The CBDV can be safely used as a dietary supplement without any adverse side effects. CBDV can be extracted from industrial hemp which is high in CBD but low in THC.

**Figure 2:**
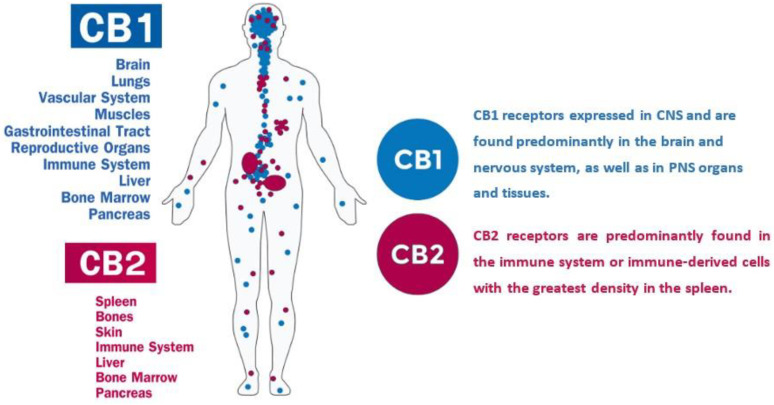
Cannabinoids receptors CB1 and CB2 and their natural distribution in the human body.

Phytocannabinoids (PCs) refer to those cannabinoid compounds synthesized in plants. There are two other different types of cannabinoids made in the mammalians called endocannabinoids (ECs), as well as synthetic cannabinoids (SCs) [82,83,84,85]. The ECs, mainly anandamide (AEA) and 2-arachidonoyl glycerol (2-AG), mediate their effects through two groups of receptors designated as cannabinoid-1 abbreviated as CB1 and cannabinoid-2 that is known as CB2 [64,86,87]. The ECs bind to CB1 and CB2 receptors with different levels of affinity. For instance, AEA has a high affinity for CB1, whereas 2-AG exhibits a higher affinity towards CB2 receptors [88]. They may also trigger multiple receptor-dependent and -independent mechanisms [81]. The CB1 receptors have been shown to be expressed in the central nervous system (CNS) and peripheral nervous system (PNS), presynaptic and axonal compartments, and also at lower levels in some extraneural tissues such as the testis, adrenal gland, bone marrow, heart, lung, prostate, thymus, tonsils, and spleen, as shown in Figure 2 [89,90,91]. They are found in particularly high levels in the neocortex, hippocampus, basal ganglia, amygdala, cerebellum, and brainstem [92,93,94]. The psycho-activity properties of cannabinoids appear to be related to these receptors. In contrast, CB_2_ receptors are primarily restricted to the PNS and mostly to the cells of the immune system with neuromodulatory functions (Figure 2) [89,90,95,96]. The mRNAs of CB2 receptors are abundant in spleen and tonsils and are found at levels equivalent to those for CB1 mRNA in the CNS [97,98].

### Phytocannabinoids Biosynthesis and Its Gene Networks

Phytocannabinoids are mainly produced in glandular hairs called trichomes [99,100,101,102], although some of the secondary metabolites such as THC and CBD have also been tracked in pollen [35], seeds [103,104], stem [105,106], leaves [73,106], and roots [106] in a low quantity [35,103,107]. However, trichomes covering the female flower are the major site of phytocannabinoids production [105,106,108].

**Figure 3:**
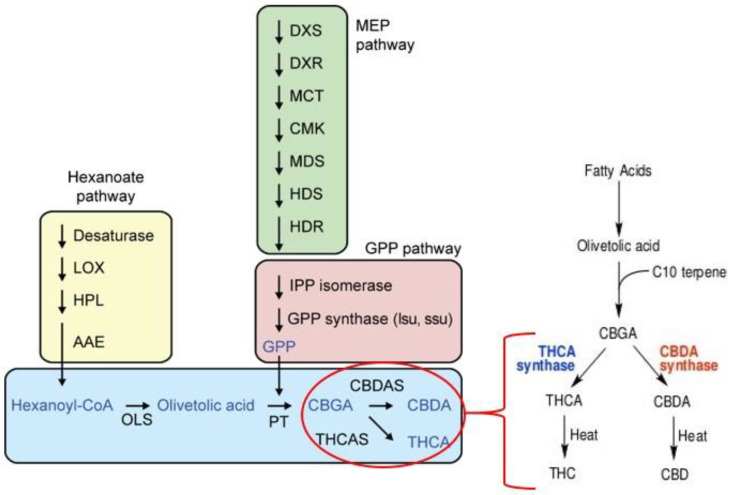
The phytocannabinoids biosynthesis pathway in cannabis and its related gene networks [12]. Heating will immediately decarboxylate THCA and CBDA into THC and CBD making them readily available for use by your body.

Cannabinoids are synthesized through either the polyketide pathway or deoxyxylulose phosphate/methylerythritol phosphate (DOXP/MEP) pathway which lead to the production of olivetolic acid (OLA) and geranyl diphosphate (GPP), respectively [109,110], and these biosynthetic pathways were well-reviewed in Flores-Sanchez and Verpoorte, 2008, Bakel et al., 2011 and Andre et al., 2016 (Figure 3). OLA production is catalyzed by polyketide synthase (PKS) and an olivetolic acid cyclase (OAC) [111]. The next stage giving rise to CBGA, the central precursor of Δ9- tetrahydrocannabinolic acid (Δ9-THCA), CBDA, and cannabichromenic acid (CBCA) includes alkylation of OLA with GPP which is catalyzed by geranylpyrophosphate:olivetolate geranyltransferase (GOT) (Fellermeier and Zenk, 1998). In the MEP pathway, however, the prenyl side-chain originates provide substrates for GPP synthesis which lead to the production of CBGA by an aromatic prenyltransferase (PT) [112]. The enzymatic reactions encompass THCA synthase (THCAS), CBDA synthase (CBDAS) and CBCA synthase (CBCAS) which convert CBGA to THCA, CBDA, and CBCA, respectively [113,114]. The last stage includes a nonenzymatic reaction during storage or smoking (heat) through decarboxylation of THCA and CBDA into THC and CBD (Figure 3) [108].

## 8. Medicinal Properties of Cannabinoids

With respect to the over one hundred phytocannabinoids found in cannabis, Δ9-THC is one of the most important psychoactive compounds [115,116]. THC is functional and effective in the human system due to its ability to mimic the skin’s natural endogenous agonists of CB receptors-endocannabinoids [81]. THC has many beneficial activities, for example, antiemetic and analgesic, and could be used for the treatment of anorexia, glaucoma, chemotherapy-induced emesis, and several types of cancer. However, its potent psychoactivity prevents or limits its medical use [117,118,119,120,121,122,123,124]. Another important promising phytocannabinoid is cannabidiol (CBD). Unlike THC, it can work independently of some cannabinoid receptors because CBD has a limited affinity for some of these traditional receptors. However, CBD has been shown to interact with other different groups of receptors. These include the peroxisome proliferator-activated receptors (PPARs), the transient receptor potential channel subfamily V member 1 (TRPV1), and the orphan G-protein coupled receptor (GPR55), and the exact roles in endocannabinoid signaling are yet to be fully clarified. CBD has anxiolytic properties which may counteract the psychoactive effects of THC [119,125].

The endocannabinoid system (ECS) is a complex cell-signaling system found in the human body which plays a regulatory role with respect to key human functions such as memory, appetite, homeostasis, and reproduction [117,126]. The ECS is made up of some key biological molecules or structures. These are the endocannabinoids (lipid-based neurotransmitters), the cannabinoid receptors and their endogenous ligands, and the enzymes mediating the synthesis and the degradation of the endocannabinoid molecules.

Apart from its neuromodulatory role, the endocannabinoid system is also involved in the regulation of energy metabolism, the body’s immune responses, and reproductive and cardiovascular processes [117,126,127,128]. Given the importance of the endocannabinoid system in the control of multiple critical processes, pharmacological intervention in order to correct disorders in processes regulated by ECS is a promising therapeutic approach. For example, cannabinoids have found useful application in the field of palliative medicine. Indeed, two cannabinoid-based drugs, nabilone and dronabinol, have now been approved for human use by the US FDA [86]. Nabilone (THC synthetic analogue) is currently used for the treatment of sleep disorders and for alleviating the symptoms of nausea and vomiting associated with chemotherapy. Dronabinol (synthetic THC) is used for treating or reversing the weight loss caused by AIDS and also for the control of nausea and vomiting associated with chemotherapy. Another drug, nabiximols (Sativex^®^, oromucosal spray, THC and CBD in 1:1 ratio), is approved for use in 10 countries in the European Union as well as Canada. In these countries, nabiximols is specifically used to treat multiple sclerosis-related spasticity. The use of cannabinoids in in vitro and animal models has indicated that these compounds exhibit anticancer effects and could potentially be used as a chemotherapeutic agent [129,130].

Among other amazing pharmacological effects of cannabinoids is related to their effects on psychiatric syndromes. The therapeutic effects of different types of cannabinoids are well known with various drugs being derived from these compounds [131]. Multiple clinical, in vivo and in vitro studies have identified the pharmacological effects as well as the positive effects of cannabinoids in psychiatric syndromes. These effects may be due to the agonistic nature of these compounds with endocannabinoids that compete with CB1 and CB2 receptors [98,132,133]. The pharmacological and therapeutic effects of endocannabinoids and phytocannabinoids are summarized in Table 1 and include antinociceptive, anti-epileptic, cardiovascular, immunosuppressive effects [134], nausea and vomiting, appetite stimulation [135], anticancer [136,137], antimicrobial [138], anti-inflammatory [139], neuroprotective antioxidants [140], and pain therapy [141]. Cannabis is also a potential drug for inflammatory bowel diseases (IBD) [142]. The other positive effects of cannabinoids have been observed in various psychiatric syndromes including depression, anxiety, and sleep disorders [143,144]. Considering unwanted side effects and other challenges, cannabinoids also could potentially be used in ophthalmology and for possible treatment of glaucoma [145]. As reviewed recently, cannabinoid can potentially be used to treat psoriasis, acne vulgaris, skin cancer, allergic contact, asteatotic and atopic dermatitis, hidradenitis suppurativa, Kaposi sarcoma, pruritus, systemic sclerosis (MS), and pathophysiology of skin inflammation, although, further approved clinical evidence must be provided to confirm their usefulness without any side effects [146,147,148].

Since the beginning of the 21st century, different countries, such as the United States, Canada, Austria, Finland, Germany, Portugal, and Spain, and just recently Iran, have authorized and regulated medical cannabis for its therapeutic effects. Some of the synthetic, extract, or natural forms of cannabinoids available on the world market or under research and clinical trials include Marinol^®^, Cesamet™, Sativex^®^, Epidiolex^®^, Bedrocan^®^, Bedrobinol^®^, Bediol^®^, Bedica^®^, Bedrolite^®^ [167,168] Namisol^®^ and Syndros^®^ (Dronabinol^®^ oral solution), Epidiolex^®^, and Arvisol^®^ (Table 2). Medical cannabis can be used in a number of different ways, for example, as pills, tablets, capsules, soft gel, dissolved in an oil solution (olive, sesame, coconut, or hempseed oil), tea, or by inhaling it after vaporization.

### 8.1. Cannabis Flos Variety Bedrocan^®^

This product contains Cannabis flos (dried female flower) with a constant composition of cannabinoids (>22% Δ9-THC and <1% CBD) and terpenes. Bedrocan^®^ is produced by the Netherlands Office of Medicinal Cannabis (OMC) under the supervision of the Ministry of Health. It was originally developed from a Jack Herer genetic background, and it is currently the most popular medicinal Cannabis variety which has been commercialized in the Netherlands [169]. Some of the therapeutic effects of Bedrocan^®^ include the treatment of spasms associated with pain in MS or spinal cord injury and myelon damage, improvement of radiation-induced nausea and vomiting, HIV medication and anorexia, chronic neurological pain, treatment of Gilles de la Tourette syndrome, and palliative/supportive care [167,170].

### 8.2. Cannabis Flos Variety Bedrobinol^®^

This product contains dried flowers of female cannabis plants with 13% Δ9-THC and 1% CBD. It has similar effects as Bedrocan^®^, except that it has a lower THC content and acts poorly. Produced by the Office of Medicinal Cannabis (OMC) in the Netherlands, it is manufactured and marketed under the supervision of the Ministry of Health and used in smokery. Some of the therapeutic effects of Bedrobinol^®^ include the treatment of spasticity associated pain in MS or spinal cord injury, the improvement of radiation-induced nausea and vomiting associated with cancers, radiation therapy and HIV therapy, all chronic pain conditions especially neuropathic pain, cachexia, anorexia in patients with cancer, AIDS, and anorexia nervosa [167,170]. Other products in this group have the same effects; however, each one contains a different composition of cannabinoids. Bediol^®^, Bedica^®^, and Bedrolite^®^ are sold as finely-ground flower heads (granules), making them easier to use. The Bediol^®^ granolate contains approximately 6.3% THC and 8% CBD. Bedica^®^ contains approximately 14% THC and less than 1% CBD. Bedrolite^®^ is composed of approximately 9% CBD and less than 1% THC.

### 8.3. Marinol^®^

This drug originated from synthetic THC and is formulated in sesame oil as oral capsules which contain Dronabinol in doses 2.5, 5, and 10 mg of active substance. It is formulated by Unimed Pharmaceuticals, Inc. US, in capsule form to increase its bioavailability and systemic uptake [167,171]. It is also marketed by Walson’s Dronabinol^®^ brand in 2.5 mg doses. Dronabinol is produced in at least six countries including South Africa under the brand name Elevat^®^. Marinol^®^ is prescribed for treating anorexia associated with weight loss in HIV/AIDS and nausea and vomiting related to chemotherapy in patients with cancer especially for those patients who do not respond to other medicines [167]. However, some side effects of Marinol^®^ include: fast heart rate or pounding heartbeat, fainting, seizure (convulsions), anxiety, diarrhea, etc.

### 8.4. Syndros^®^ (Dronabinol^®^ Oral Solution)

Dronabinol oral solution was formulated and approved with a maximum dronabinol dose of 5 mg to solve the problem of patients with a hypersensitivity to sesame oil. Syndros^®^, however, is contraindicated in patients with hypersensitivity to alcohol, or with recent use of disulfiram- or metronidazole-containing products.

### 8.5. Namisol^®^

Namisol^®^ is a new formulation of a tablet containing purified Δ9-THC (>98%) in fixed dosages with high and predictable bioavailability and beneficial pharmacokinetic and pharmacodynamics characteristics after oral administration [172]. Echo Pharmaceuticals BV has been formulated Namisol^®^ using a patented drug delivery technology, Alitra™, to improve absorption and distribution of compounds with low water solubility. The Namisol^®^ was developed to improve abdominal pain resulting from chronic pancreatitis (CP).

### 8.6. Cesamet™

This product is a synthetic derivative of THC in the form of oral capsules (purple and white) which contain 1 mg (2.7 µmol) nabilone. Chemically, nabilone is similar to the Δ9-THC occurring in *Cannabis sativa* L. The drug is formulated by Valeant Pharmaceuticals International, USA and is used in the treatment of nausea and vomiting caused by cancer chemotherapy [167] or may be effective in chronic noncancer pain [173]. It has complex effects on the CNS through CB1 receptors [173].

### 8.7. Sativex^®^

Perhaps Sativex^®^ is the most famous authorized product derived from cannabis to date which is a natural extract of cloned phenotype is the form of mouth spray. Sativex^®^ is formulated by GW Pharm Ltd., in Canada, and its active substance, Nabiximols, contains 2.7 mg of Δ9-THC and 2.5 mg of CBD in a 1:1 ratio. Sativex^®^ is prescribed in MS patients to relieve muscle stiffness and neuropathic pain and sleep disturbances [167,174]. The most common adverse effects of Sativex^®^ include: dizziness, sleepiness, fatigue, feeling of intoxication, and a bad taste. Satinex^TM^ is an analog formulated by Yasdaru Pharmaceutical Inc. Iran, in the form of buccal spray using a natural extract of native cloned phenotype which is obtained via the Accelerated Solvent Extractor (ASE) method, and its active substance contains 2.7 mg of Δ9-THC and 2.5 mg of CBD in a 1:1 ratio.

### 8.8. Epidiolex^®^

This product is the first FDA-approved prescription plant-derived, purified pharmaceutical-grade cannabidiol (CBD), which is administered orally to treat seizures associated with Lennox-Gastaut syndrome or Dravet syndrome [175]. Epidiolex^®^ contains no Δ9-THC.

### 8.9. Arvisol^®^

This product is the world’s first pure cannabidiol tablet which contains pure CBD in fixed dosages with high and predictable bioavailability. Identical to Namisol^®^ (oral tablet containing pure THC), Arvisol^®^ (oral tablet containing pure CBD) is formulated by Echo Pharmaceuticals BV using its patented technology on drug formulation, Alitra™.

There are several other pure CBD products in development. CBD gel (ZYN002) is designed by Zynerba^®^ Pharmaceuticals and is intended to be registered for transdermal use, Fragile X syndrome, adult refractory focal epilepsy, and encephalopathies that are developmental. Bionorica^®^ has developed a pure CBD crystalline powder from certified seeds of low-THC fiber hemp. Synthetic CBD crystalline powder is also available as an oral capsule formulated by STI Pharmaceuticals along with other forms of products such as CBD oil and inhalation formulation [176,177,178]. Oral solutions of pure CBD in different doses are under clinical trial studies by INSYS Pharmaceuticals, for childhood absence seizures, or as an adjunctive therapy in conjunction with vigabatrin for infantile spasm-type seizures, Prader-Willi syndrome, and anxietylike behavior [179]. Purified CBD is also formulated in seamless gelatin matrix pellets in a dose of 10 and 100 mg CBD (PTL101), by PhytoTech Therapeutics [180].

## 9. Quality for Health and Nutrition: Cannabis as Food and Feed

Over the past decade, the scientific community and public society have increasingly accepted cannabis, specifically hemp, as much more than a recreational drug. There is still a lack of awareness and studies and much confusion in the distinction between industrial cannabis/hemp, medicinal cannabis/hemp, and drug-type cannabis. Hemp varieties contain THC level less than 0.2–0.3% of the reproductive part of the female plant at flowering. Various parts of the hemp plant, seeds, extract, and oil, and also the cannabis plant with acceptable lower levels of THC, can also be used as drinks, a perfect nutritional dietary supplement, or as a dry super food in times of crisis such as floods, earthquakes, wars and quarantines, and as animal feed. The variety of THC products is less than the CBD products due to its limitations, but still some products such as cannabis tea (also known as weed tea, pot tea, ganja tea, or a cannabis decoction), THC coffee, and gums and cookies are sold on the market. Hemp has many applications and can be used for the production of fiber, clothing, paper, textiles, rope, biodegradable products, hempcrete, paint, lubricants, biofuel, flour and oil, food, lotions, shampoos, cosmetics, drinks, animal feed, organic compost, and mulch [181,182,183]. Hempseed can be consumed as whole, hulled seed, or dehulled (hempseed kernel), oil, flour, and protein powder [183]. Hempseed, hempseed oil, hemp snacks, and hemp protein have been considered as major sources of healthy ingredients and most nutritionally complete food [181]. Hempseed contains 20–30% carbohydrates, 20–25% proteins (20 amino acids that the body cannot produce itself), polyunsaturated fatty acids (PUFAs) especially omega-3 and 6 (ideally 3:1 ratio), soluble and insoluble fiber, minerals (zinc, potassium, calcium, phosphorus, iron, magnesium, manganese, and selenium), vitamins (A, B complex, C, and E), antioxidants, carotenoids, phytosterols, flavonoids, phenolic compounds, terpenes, and terpenoids [183,184,185].

Hempseed oil is composed of polyunsaturated fatty acids known for their beneficial effects against cardiovascular diseases, cancer, and inflammatory conditions [186]. Over 189 lipids, including 52 phospholipids and 80 sulfolipids [187] and 147 compounds belonging to flavonoids, proanthocyanidins, and phenolic acids [188] have been identified in hemp. Hemp sprouts as food known are for their positive cardiovascular and metabolic effects, and contain a higher content of total polyphenols, flavonoids, and flavonols compared to hempseeds [189]. In animal diets, essential oils (EOs) play an important role as an alternative to antibiotics [190,191]. Bioactive ingredients such as EOs, herbs, plant extracts, and seeds are usually used in veterinary medicine for better performance and yield enhancements [192]. All these positive effects are accompanied by the fact that “you cannot get high from CBD”.

## 10. Current and Future Trends for Cannabis/Hemp Products

Whether hemp originated from wild drug-type cannabis and/or marijuana evolved from hemp, is an issue that requires much more accurate scientific evidence. What is clear, however, is that humans and cannabis both have experienced a long history of thousands of years with many ups and downs. Cannabis/hemp is considered among the most ancient cultivated plants with many secrets. There is still controversy over the origin of cannabis, but what is clear is that, due to its versatile capabilities, this plant has spread all over the world from central Asia to Europe, America, Africa, and Australia due to the fact that cannabis/hemp is a multipurpose, sustainable, and low environmental impact crop which can be useful for several applications in agricultural, food and feed, cosmetic, building, pharmaceutical, and military industries [183].

What the international community is witnessing right now is an effort to adjust countries’ policies and to reach a consensus on cannabis/hemp to have the maximum productivity of this plant. Especially over the past decade, the scientific community and public society have increasingly accepted cannabis specifically hemp as much more than a recreational drug. In fact, the introduction of hemp as a valuable promising source with medicinal and nutritional properties has somewhat alleviated public concerns. However, there is still a lack of awareness and much confusion in the distinction between different types of cannabis. Whether it is accepted or not, the current trend is to improve the value of hemp and its various derived products so that it becomes more visible in human life, while respecting concerns, laws, and regulations.

Among hundreds of products derived from cannabis, there are growing demands for cannabinoids, mainly CBD, with many diverse therapeutic and nutritional properties in veterinary or human medicine. Today, more and more people around the whole world tend to use different forms of cannabinoids, and there is fierce competition between companies to formulate and receive approval for new products, which indicates that a large part of CBD’s immediate future is the pharmaceutical and food industries. Towards such disciplines, more countries are rapidly adapting regulations to accommodate these new uses, despite the limitations related to THC. The output of such an idea would be that the perception of cannabis as an abuse drug that harms people is progressively changing to cannabis/hemp as a medicine, food, feed, drink, beauty, and health product. Hemp potential as source of supplementary and healthy food remains largely to be investigated in adequately powered clinical trials. However, still the biggest challenges towards acceptance and consensus of such an idea which must be addressed will be THC content, public concerns, lack of knowledge and awareness, gaps in data, ethnic and religious prejudices, national or regional regulations and regulatory authorities, FDA concern regarding health risk assessment, and significant investment of capital and resources.

People around the world are consuming a diverse range of CBD products either within the authorized or unauthorized framework; however, CBD has not yet been approved neither as feed nor food in many regions such as EU and Iran; although, medical hemp will be authorized in Iran. In this regard, breeding new hemp varieties with low THC (<0.2%–0.3%) and high CBD content from different genetic resources and growing superior industrial varieties under controlled conditions, will pave the way for easier and faster acceptance of this issue.

In case of the review and rewriting of the national regulations related to cannabis, Iran can be considered among the best ideal regions for the outdoor growing of cannabis and hemp using original native varieties based on our experience over the past decade. As a snack or bird food, hempseed has long existed in Persian culture. Cannabis is called “Shah-Daneh” in Persian, which means “king of the seeds” and has been cultivated for many years. Hempseed nutritional properties have not really been recognized in Iran as food for humans and feed for domesticated animals. However, a redoubled effort has been made over the past decade to introduce hempseed as food and feed.

## 11. Conclusions

The important role of medicinal herbs in the prevention and treatment of many diseases throughout the history of human life is inevitable. Extensive surveys of historical evidence, very diverse genomes, and a plethora of unique medicinal and industrial properties found in cannabis suggest a very long history of humans using cannabis, although with many ups and downs, mainly because of THC. However, with the emergence of the hemp and CBD market, consisting of products both in the food supplement and medicine sectors, the chain’s value has been improved, and CBD has recently become the face of the cannabis industry, due to THC limitations and new discoveries about medicinal properties of CBD and other natural rare cannabinoids. Now, it is our turn to get cannabis high. Phytocannabinoids are not the only valuable components found in *C. sativa*. Phenolic compounds, terpenoids, alkaloids, and noncannabinoid phenols are some of the secondary metabolites present in this plant. Here, however, we tried to summarize current findings concerning natural phytocannabinoids mainly THC and CBD and some other synthetic forms of cannabinoids available on the market and their therapeutic properties towards putting these valuable compounds into food and health baskets.

Today, there are growing demands for compounds generally called “cannabinoids” with many diverse therapeutic and nutritional properties, and more and more people and patients from different countries are consuming such products either within the framework of a precise medicine or outside of this framework. However, the international community has not yet reached a consensus on cannabis and the multiple potential advantages; marketing of CBD-based dietary supplements is under intense debate in the scientific community. In recent years, however, the introduction of hemp as a valuable promising source with medicinal and nutritional properties and the emergence of CBD in medicine have somewhat alleviated these concerns. Hemp products can be used as a nutritional supplement for the supportive care of patients or even as a super food in times of crisis such as floods, earthquakes, wars, and quarantines. Despite all concerns regarding cannabis, nobody can ignore the use of cannabinoids as promising tonic, analgesic, antipyretic, antiemetic, anti-inflammatory, anti-epileptic, anticancer agents, which are effective for pain relief, depression, anxiety, sleep disorders, nausea and vomiting, multiple sclerosis (MS), cardiovascular disorders, appetite stimulation and reversing the weight loss in AIDS and chemotherapy.

There are still many secrets in cannabis/hemp that need to be discovered, and this plant deserves to be nominated as a candidate to save humanity as a promising source of medicine, food, and feed in the difficult future ahead. Even though this review is focused on cannabinoids and their possible impacts on health, it also highlights the lack of data and a great gap between real cannabis values and public acceptance. In this regard, creating a worldwide network to gather all cannabis experts together as the “Cannabis World Union” is proposed to better think about this magic plant and to avoid sowing confusion whether in the medical and scientific community or in public society. This is exactly what we are trying to achieve at MedCannaBase (www.medcannabase.org).

## Figures and Tables

**Table 1:** Pharmacological and therapeutic effects of endocannabinoids and phytocannabinoids.

Effects	THC	AEA	2-AG	CBD	CBC	CBDV	CBG	THCV	References
Suppress Seizure								√	[149]
Reduce Parkinson’s Disease symptoms								√	[150]
Anti-inflammatory		√	√	√	√	√	√	√	[127,128,151,152,153,154]
Reduce weight								√	[155]
Anticonvulsant						√			[156]
Anxiolytic				√					[119,125]
Effective against cancer	√	√	√	√			√		[120,123,124,127,128,157,158]
Chemotherapy induced emesis	√			√					[117,122,159]
Effective against glaucoma	√	√					√		[121,127,128]
Epilepsy				√		√			[151,160]
Oxidative injury				√					[156]
Schizophrenia				√			√		[161,162]
Neuro-degeneration				√					[163]
Diabetic retinopathy				√					[164]
Colitis							√		[77]
Exhibits Neuron protection					√				[165,166]
Antiemetic, Analgesic activity	√				√				[119]
Energy and fat metabolism		√	√						[127,128]
Insulin sensitivity		√	√						[127,128]
Anorexia	√								[117,118]
Bowel disease							√		[165]

**Table 2:** Summaries of different forms of cannabinoids available on the world market or under development and clinical trials.

Product	Possible Therapeutic Effects	Component	References
Bedrocan^®^	✓ Spasms associated with pain in MS ✓ Myelon damage ✓ Improvement of radiation-induced nausea and vomiting HIV medication and anorexia ✓ Chronic neurological pain ✓ Treatment of Gilles de la Tourette syndrome palliative/supportive care	(>22% Δ9-THC and <1% CBD)	[167,170]
Bedrobinol^®^	✓ Treatment of spasticity associated pain in MS ✓ The improvement of radiation-induced nausea and vomiting associated with cancers, radiation therapy and HIV therapy ✓ Chronic pain conditions especially neuropathic pain, cachexia, anorexia in patients with cancer, AIDS, and anorexia nervosa	13% Δ9-THC and 1% CBD	[167,170]
Marinol^®^	✓ Treating anorexia associated with weight loss in HIV/AIDS ✓ Treating nausea and vomiting related to chemotherapy in patients with cancer especially for those patients who do not respond to other medicines	Dronabinol in doses 2.5, 5, and 10 mg	[167,171]
Namisol^®^	✓ improve abdominal pain resulting from chronic pancreatitis (CP)	Purified Δ9-THC (>98%)	[172]
Cesamet™	✓ Treatment of nausea and vomiting caused by cancer chemotherapy ✓ Effective in chronic noncancer pain [178]	THC in the form of oral capsules which contain 1 mg (2.7 µmol) nabilone	[167,173]
Sativex^®^	✓ To relieve muscle stiffness and neuropathic pain and sleep disturbances in MS patients	Nabiximols contains 2.7 mg of Δ9-THC and 2.5 mg of CBD in a 1:1 ratio. Sativex^®^	[167,174]
Epidiolex^®^	✓ To treat seizures associated with Lennox-Gastaut syndrome or Dravet syndrome	Purified pharmaceutical grade cannabidiol (CBD),	[175]
